# Efficient promiscuous Knoevenagel condensation catalyzed by papain confined in Cu_3_(PO_4_)_2_ nanoflowers†

**DOI:** 10.1039/c7ra12940h

**Published:** 2018-01-09

**Authors:** Jianyun Yu, Xinxin Chen, Min Jiang, Anming Wang, Linlin Yang, Xiaolin Pei, Pengfei Zhang, Stephen Gang Wu

**Affiliations:** College of Materials, Chemistry and Chemical Engineering, Hangzhou Normal University Hangzhou 310014 P. R. China waming@hznu.edu.cn +86-571-28865630 +86-571-28865978; Department of Energy, Environmental and Chemical Engineering, Washington University St. Louis MO 63130 USA

## Abstract

To develop an efficient and green immobilized biocatalyst for promiscuous catalysis which has a broad scope of applications, hybrid nanoflower (hNF) confined papain as a biocatalyst has been proposed and characterized in this study. hNFs were firstly prepared through mixing CuSO_4_ aqueous solution with papain in phosphate saline (PBS) at room temperature. The resulting hNFs were characterized by SEM and verified through a hydrolysis reaction with *N*-benzoyl-dl-arginine amide as substrate. Under optimal conditions, this nano-biocatalyst demonstrated a 15-fold hydrolytic activity compared with papain of free form, along with better thermal stability. A series of reaction factors (reaction temperature, time, and solvent) have been investigated for Knoevenagel condensation reactions with hNFs as catalyst. At optimal conditions, product yield of the hNFs catalyzed reaction was 1.3 fold higher than that of the free enzyme with benzaldehyde and acetylacetone as substrates. A few aldehydes and methylene compounds have also been used to test the generality and scope of this new enzymatic promiscuity. To sum up, the obtained hNFs demonstrate better catalytic properties than free papain and the inorganic metal-salt crystal can function as both support and promotor in biocatalysis.

## Abstract 2

Knoevenagel condensation was catalyzed and enhanced by Cu^2+^ and papain on hybrid nanoflowers (hNFs) in the promiscuous catalysis.

## Graphical abstract

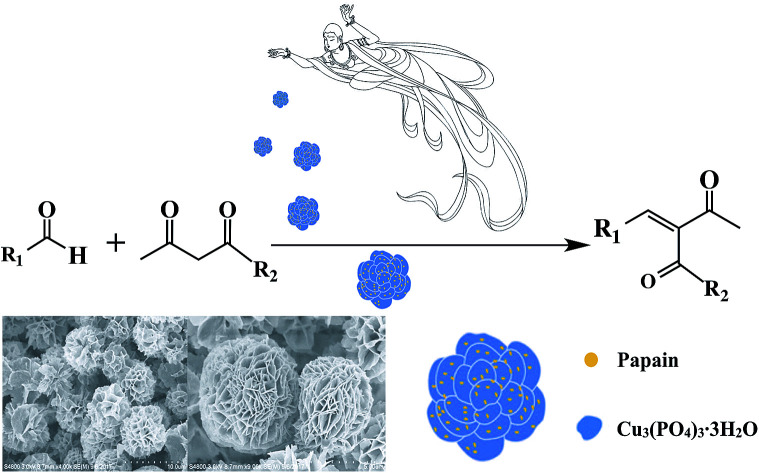

## 1. Introduction

Knoevenagel condensation^1,2^ is a common and versatile method for C–C bond formation and widely used in both academic research and chemical industries for the preparation of fine chemical intermediates, polymers, cosmetics, perfumes, and therapeutic drugs such as antibody–drug conjugates (ADCs).^3^ This reaction can be catalyzed by organo-bases,^4^ such as pyridine or piperidine.^5^ Homogeneous base catalysis is often time-consuming, because acid has to be added to neutralize the waste afterwards. Further, undesired side-reactions such as oligomerization may occur with relatively low reaction temperatures, and catalyst recovery is challenging.^6^ As reported, the Knoevenagel condensation reaction^2,7,8^ can take place on ethyl malonate (as substrate) bound to the Wang resin, however, product cleavage requires trifluoroacetic acid, leading to excess pollution when it afforded pure coumarin-3-carboxylic acid derivatives.^9^

To overcome those drawbacks mentioned above, numerous efforts have been made to develop better heterogeneous Knoevenagel catalysts, for example, metal-salt, nanoparticle catalyst, modified zeolites, ionic liquids or magnetic base analogues.^6,10,11^ Among them, supported metal and metal-salt catalysts attract much attention because they can be easily recovered from the reaction mixture and reused in the next cycle.^12^ This recyclable heterogeneous Pd catalyst can partially resolve some issues of homogeneous Pd ligands catalyst in Knoevenagel condensation.^13,14^ However, as a transition metal element, Pd is expensive and the preparation of this supported catalyst is time-consuming and not environmentally friendly. In comparison, copper is much more preferred in terms of cost and availability: copper powder can also catalyze the condensation of cyanoacetate and benzaldehyde under mild conditions.^6^ Further, copper salt provides better catalytic activities compared metal form and is considered as a green and effective catalyst for organic reactions. For instance, Zhou *et al.* has developed a procedure for synthesis of substituted benzimidazo[1,2-*a*] quinolones *via* Knoevenagel condensation and intramolecular Ullmann-type coupling by using CuI as catalyst, with a reaction condition of 5–7 hours at 90 °C and a yield of 84%.^15^ In general, most organic synthesis using metal or metal salt as catalysts requires shorter reaction time at the cost of higher reaction temperature.^16–20^

In comparison, papain acting as a promiscuous biocatalyst has been reported to catalyze Knoevenagel reactions in DMSO/water and is considered as a sustainable and inexpensive option.^21^ Promiscuous catalysis is defined as catalyzing more than two kinds reactions simultaneously, which is different from substrate promiscuity. The difference lies in the fact that the latter requires stabilization of different transition state in the reaction process.^22,23^ Catalytic promiscuity has been widely employed in industrial biocatalysis and organic synthesis, because it expands the genre of practicable reactions along with advantages of enzymatic catalysis such as mild reaction conditions, simple separation, and high yields.^24^ Further, papain from *carica papaya* has been reported to present catalytic promiscuity and catalyze the synthesis of trifluoromethyl carbinol derivatives *via* aldol reaction between α,α,α-trifluoromethyl ketones and aliphatic ketones.^22^ Moreover, Wei and coworkers have studied the possible reaction pathway for papain-catalyzed hydrolysis of *N*-acetyl-Phe-Gly 4-nitroanilide (APGNA).^25^ The whole hydrolysis process includes two stages of acylation and deacylation. However, application of enzymatic promiscuity is challenged by low activity of enzymes in promiscuous reactions. A type of enzyme catalyst with high catalytic activity in enzymatic promiscuous reactions would greatly expand the application spectrum of enzyme promiscuity. It will be very much desirable if metal salt with catalytic superiority can be combined with immobilized enzyme.

In this work, we propose to combine the advantages of enzyme promiscuity biocatalysis with the strengths of metal salt catalysis in Knoevenagel condensation. Inorganic Cu salt crystal has the potential to work as both chemical catalyst and support simultaneously. For instance, promiscuous reactions can be catalyzed by papain attached on inorganic Cu salt crystal. Cu_3_(PO_4_)_2_·3H_2_O was firstly generated in papain aqueous solution, then papain was embedded to form papain-inorganic hybrid nanoflowers. Its catalytic properties were characterized *via* Knoevenagel condensation under mild reaction conditions. Further, we also determine its catalytic generality and scope through a few aldehydes and methylene compounds.

## 2. Materials and methods

### 2.1 Materials

Papain (activity ≥ 400 U mg^−1^) was purchased from Shanghai Tripod Biological Technology Ltd. Dimethyl sulfoxide was purchased from Sangon Biotech and acetaldehyde (40%) was purchased from Tianjin Guangfu Chemical Industry Research Institute. Phenyl acetaldehyde (95%), nitrobenzene formaldehyde (97%), 4-bromobenzaldehyde (99%), magnesium sulfate anhydrous, *N*-alpha-benzoyl-l-arginine, and *N*-benzoyl-l-arginine ethyl ester hydrochloride (BAEE) were purchased from Aladdin (Shanghai). 4-Nitroaniline and *N*-benzoyl-dl-arginine amide hydrochloride (BAPNA) were purchased from Sigma. Benzoyl acetone was purchased from Alfa Aesar chemistry Ltd (Tianjin). Ortho nitrobenzene (99%) was purchased from Damas-beta Chemistry Ltd. 4-Chlorobenzene formaldehyde (98.5%) was purchased from Acros Organics. All other chemicals were provided by Sinopharm Chemical Reagent Ltd (Shanghai).

### 2.2 General procedure of hybrid nanoflowers-catalyzed Knoevenagel condensation

**Fig. 1:**
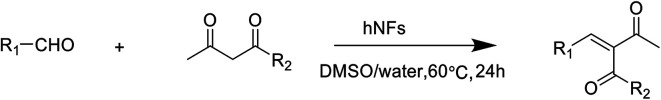
The Knoevenagel condensation reaction catalyzed by hNF (R_1_, phenyl; R_2_, methyl).

Typically, benzaldehyde (212 mg, 2 mmol) and acetylacetone (240 mg, 2.4 mmol) were added to a 10 mL of round bottom flask. Subsequently, DMSO (3.75 mL), DI water (1.25 mL), and papain-inorganic hybrid nanoflower (containing 150 mg papain) were added in this sequence. This mixture was shaked at 150 round per minute and incubated at 60 °C for 24 h (Fig. 1) and monitored by thin chromatography (TLC). After that, the reaction was terminated by filtering out enzyme. The filtrate was washed twice with DI water (5 mL) and diluted with ethyl acetate (10 mL). The remaining aqueous phase was extracted with ethyl acetate until most organic matter was extracted from aqueous phase. The solution was dried through adding anhydrous MgSO_4_ and the filtrate was obtained through filtering and purified *via* flash column chromatography (ethyl/petroleum ether).

### 2.3 Preparation and characterization of papain-inorganic hybrid nanoflowers

**Fig. 2:**
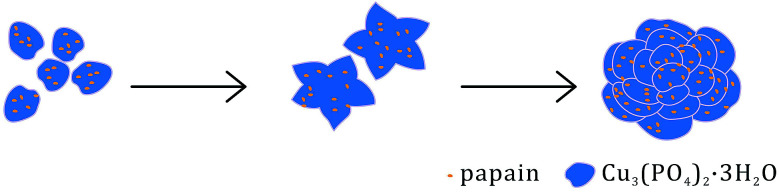
Schematic forming process of hNFs.

The papain-inorganic hybrid nanoflowers were synthesized following procedures reported:^23,26^ 20 μL of CuSO_4_ solution (120 mM) was added to 3 mL of phosphate buffered saline (PBS) solution (10 mM) containing 0.25 mg mL^−1^ papain at pH 9.0 (Fig. 2). This mixture was then kept at room temperature (∼25 °C) for 3 days and centrifuged at 5000 rpm for 5 min (4 °C). The resulting precipitation was kept and washed with DI water for three times.

### 2.4 Activity assay of papain preparations

Two methods were employed to test the activity of papain preparations^27,28^ and to determine the catalytic properties of hNFs. In this first method, BAPNA was used as substrate, enzyme liquor (100 μL) was firstly added into a 10 mL plastic pipe and placed in water bath (40 °C) for 5 min. After that, BAPNA (0.4%, 2 mL) was added in and OD_405_ was determined after 10 minutes. In the second method, BAEE was used as the substrate. Enzyme liquor (100 μL) was also added into 10 mL plastic pipe and placed in water bath at 37 °C for 5 min. Afterwards, BAEE (0.4%, 2 mL) was added in and mixed, OD_253_ was examined after 10 minutes. In both methods, substrate solutions were used as blank control.

### 2.5 Characterization of products in promiscuous catalysis

^1^H NMR spectra and ^13^C NMR were recorded through a Brucker DPX 300 spectrometer at 500 MHz and 126 MHz, respectively. NMR experiment was performed in deuterated chloroform (CDCl_3_) containing proper concentration of sample, a total 128 scans were recorded. Data was collected and analyzed through MestReNova software. Proton chemical shifts were referenced to tetramethylsilane (TMS) at 0.00 pm. Scanning electron microscope (SEM) (S-4800, HITACHI) was employed to examine the morphology of hNFs and Gas Chromatography Mass Spectrometer (GC-MS, Agilent 5975) was used to measure the molecular weight of products.

## 3. Results and discussions

### 3.1 Characterization of papain-inorganic hybrid nanoflower

**Fig. 3:**
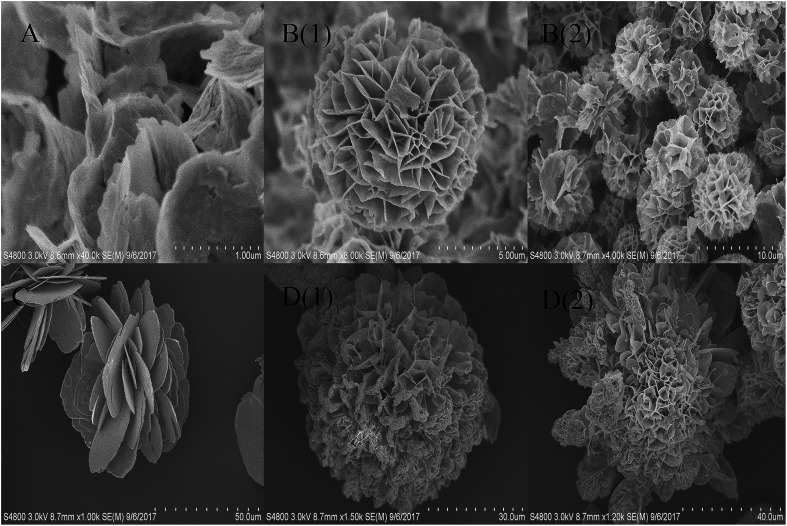
SEM image of papain nanoflowers synthesized with 0.25 mg mL^−1^ papain at different PBS; (a) PBS (200 mM, pH 5.5); (b) PBS (200 mM, pH 7.0); (c) PBS (200 mM, pH 9.0); (d) PBS (100 mM, pH 7.4).

PBS solution of different concentrations (4, 20, 100, and 200 mM) and pHs (5.5, 7.0, and 9.0) to examine the hNFs morphology and structure. As shown in Fig. 3 of SEM graphs of papain-inorganic hybrid nanoflowers, the size of hNF was similar to those previously reported.^29–31^ From SEM images, petal cannot grow when the concentration of buffer was low (*e.g.*, 4 or 20 mM), and was only observed on the surface of Cu_3_(PO_4_)_2_ crystals at this condition. With the increase of PBS concentration, nanoflowers can be much more easily observed. The size of flower synthesized at 100 mM PBS was larger than at 200 mM (Fig. 3b and c). Petals cannot be observed only if the pH of PBS reached certain level (*e.g.*, 7.0 and 9.0). For catalytic hydrolysis reaction in the activity characterization, the prepared hNFs demonstrate about 15 folds of activity higher than that of free papain with either substrate. For kinetics property of the enzyme preparations, the results were shown in Table 1. It was found that the *K*_m_ value of free papain was smaller than hNFs', which mean that the hNFs had a better affinity for the corresponding substrate. In addition, the *K*_cat_/*K*_m_ value of hNFs was higher than that of free enzyme, which showed the reaction rate of former enzyme preparation was better.

**Table 1:** The kinetics parameter of free enzyme and hNFs

	*K*_m_ (mM)	*K*_cat_ (s^−1^)	*K*_cat_/*K*_m_ (mM^−1^ s^−1^)	*V*_max_ (mM min^−1^)
Papain	1 ± 0.35	2.67 ± 0.55	2.67 ± 0.59	12.17 ± 0.93
Papain-hNFs	0.34 ± 0.02	1.03 ± 0.04	3.06 ± 0.1	15.27 ± 0.23

During the formation process of hNFs, Cu^2+^ was proposed to firstly react with phosphate to generate primary copper phosphate complexes. Subsequently, the amine groups from papain backbone were coordinated with copper phosphate to initiate the nucleation of nanocrystals. Large agglomeration of protein molecules and primary crystals were formed afterwards. The growth of copper phosphate crystals originated at the individual Cu^2+^ binding sites on the surfaces of the agglomeration, leading to the appearance of separate petals and finally the formation of flower-like structure. The fact that papain can co-crystallize with Cu_3_(PO_4_)_2_·*x*H_2_O was possibly driven by relatively high affinity between amine groups of enzyme backbone and Cu^2+^. With the growth of hybrid nanoflowers, the morphology of hybrid nanoflowers also became complete and compact.

### 3.2 Effect of reaction temperature on 3-benzylidenepentane-2,4-dione yield in promiscuous catalysis

**Fig. 4:**
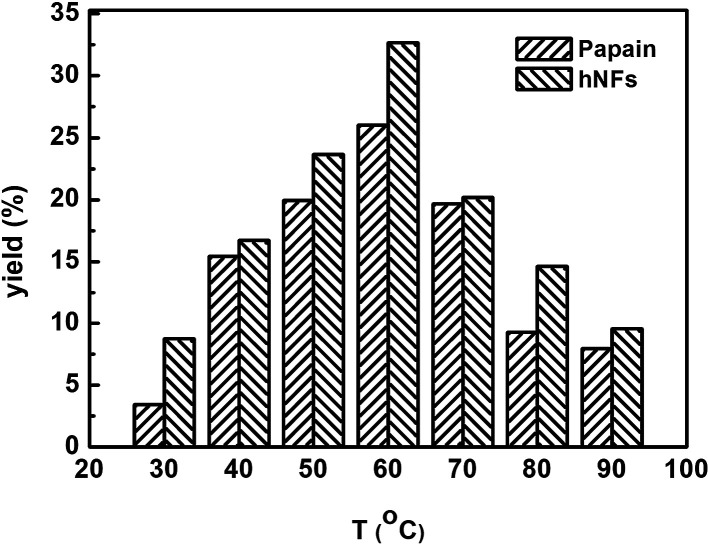
Effect of reaction temperature on 3-benzylidenepentane-2,4-dione yield in promiscuous catalysis.

Fig. 4 shows the effect of reaction temperature on product yield in promiscuous catalytic Knoevenagel condensation. The optimum product yield reached 32.67% when hNFs was used as catalyst, 1.25 folds of that was obtained when papain was used as catalyst. Moreover, under other reaction temperatures, product yields catalyzed by hNFs were also higher than that of papain. In hNFs, papain was confined within Cu_3_(PO_4_)_2_·*x*H_2_O, which stabilized enzyme protein under different reaction temperatures. For copper phosphate, Cu^2+^ is able to stabilize the enzyme structure by its complexation with the amine groups in the backbone of papain and accelerate the reaction process in promiscuous catalysis.^11^

### 3.3 Effect of reaction time on 3-benzylidenepentane-2,4-dione yield in promiscuous catalysis

**Fig. 5:**
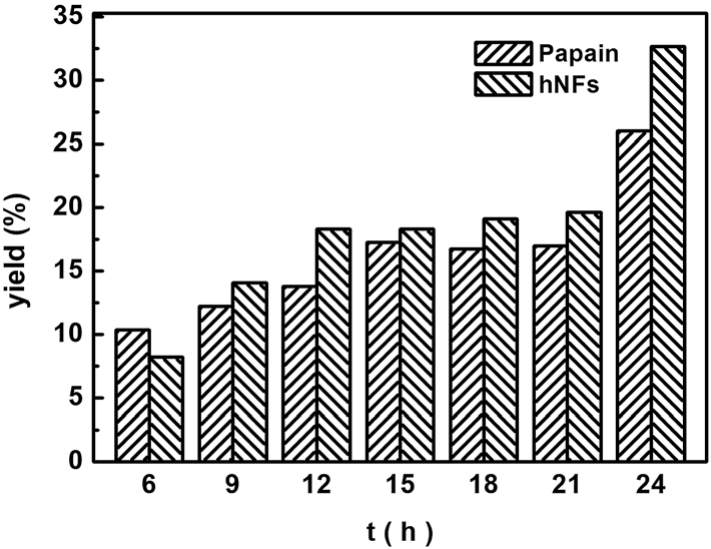
Effect of reaction time on 3-benzylidenepentane-2,4-dione yield in promiscuous catalysis.

The effects of reaction time on the Knoevenagel condensation were determined through reactions at 60 °C for different periods, ranging from 6 h to 24 h (Fig. 5). After 6 h, hNFs catalyzed reaction demonstrated higher yields compared with free papain. At 6 h, papain catalyzed reaction has a higher product yield higher than hNFs, which can be explained by improved mass-transfer limitations between enzyme and substrate after immobilization.^32^ It takes some time for hNFs to achieve its maximal catalytic efficiency. The yield reached up to 32.67% after 24 h with hNFs. Moreover, with additional reaction time after 24 h, the product yield will still increase, presenting desiring thermostability. In a nutshell, the catalytic activity of hNFs is better than papain in terms of product yield.

### 3.4 Effect of different media on 3-benzylidenepentane-2,4-dione yield in promiscuous catalysis

**Fig. 6:**
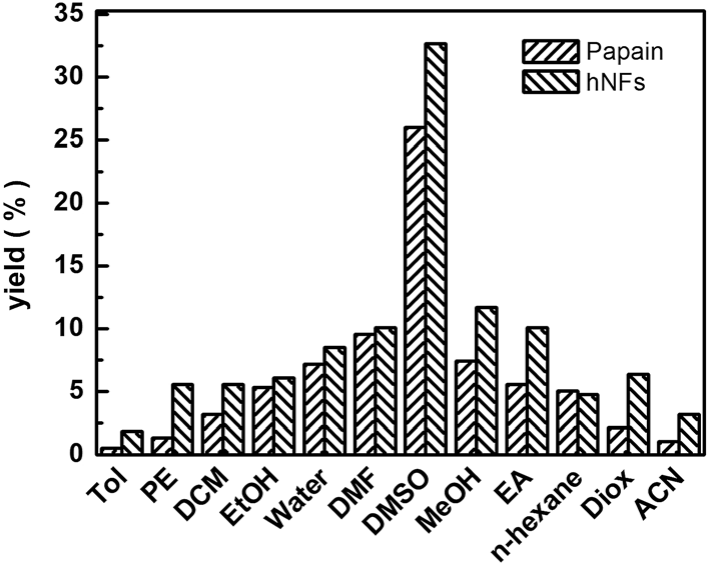
Effect of solvent on 3-benzylidenepentane-2,4-dione yield in promiscuous catalysis.

The effect of media on promiscuity catalysis was shown in Fig. 6. Best yield was reached when DMSO was used as solvent, which is associated with its good solubility. In addition, the solvent may have an effect on the conformational of enzyme which was likely to be the main influence factor of catalytic activity. The minimal changes of the enzyme structure and conformation had an effect on the great change on the interaction between enzyme and substrate possibly, which consequently result in sharply increasing or decreasing of the reaction rate. The hNFs of papain were shown to undergo conformational changes, including rearrangement of surface amino acids, stripping of water from the protein surface, and weakening of hydrophobic interactions when exposed to organic solvent.^33^ In our experiments, the result of catalysis showed that the hNFs enzyme preparation was organic solvent-tolerant. Using polar solvents such as DMSO and protic solvents such as ethanol and methanol always lead to higher yields. In contrary, less polar solvents such as toluene are not good media for Knoevenagel condensation.^21^ Further, the effect of other solvents was negligible compared with DMSO. For instance, the yield was up to 32.67% when solvent was DMSO, however, the yield only reached 1.86% when toluene was used as solvent for hNFs. The results are consistent for either catalyst (hNFs or papain). Moreover, the yield was unremarkable when hexane and DMF were used as solvent.

### 3.5 Effect of water content and reuse cycle times on 3-benzylidenepentane-2,4-dione yield in promiscuous catalysis

**Fig. 7:**
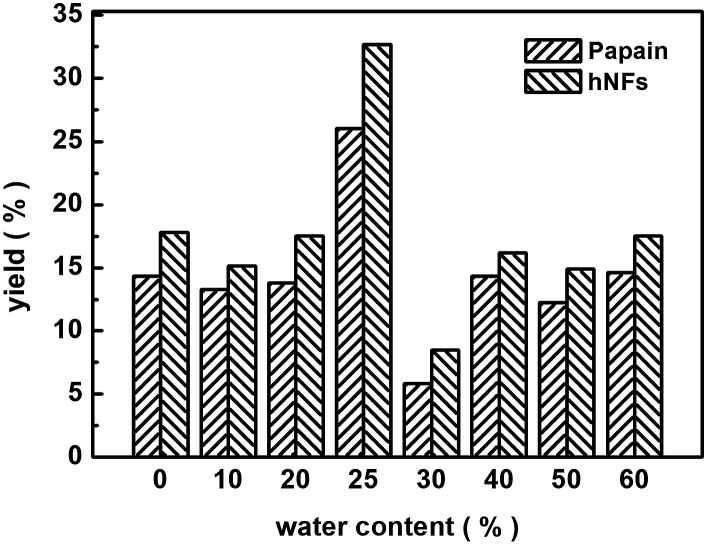
Water content on 3-benzylidenepentane-2,4-dione yield in promiscuous catalysis.

Fig. 7 displays the effect of water content in media on the yield of promiscuous catalysis. The highest yield was reached when the water content was 25%, whether catalyzed by free papain or hNFs. This observation can be potentially explained by interactions between water and solvent.^21^ There is a point that the interaction between water and solvent contributed maximal effects on promiscuous catalysis. Water can play a “lubricant” role in keeping the conformation of enzymatic activity in organic solvent, therefore, is required in promiscuous catalysis.^21^

**Fig. 8:**
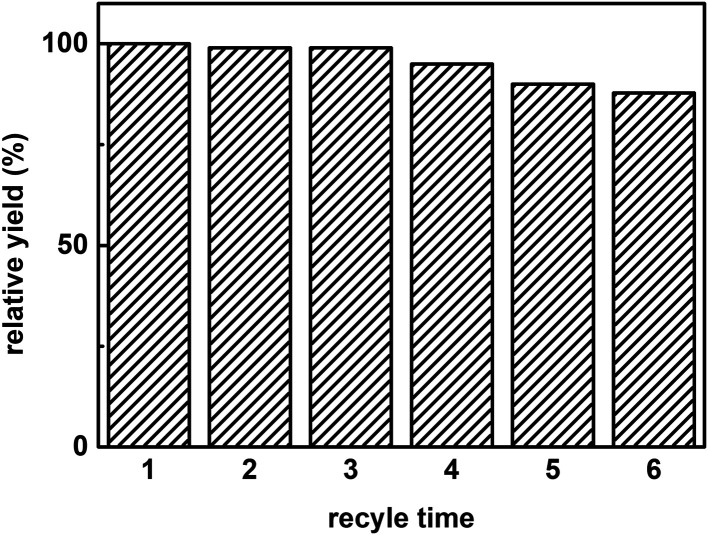
The recycle time on promiscuous catalysis.

Fig. 8 shows the reuse cycle time in promiscuous catalytic synthesis of 3-benzylidenepentane-2,4-dione in organic solvent and the product 3-benzylidenepentane-2,4-dione yield every time. The synthetic yield of this product at first time was set as 100%. It was obvious that the stability of hNFs was good due to the result that the yield was almost constant after cycling for 3 times. The catalytic synthetic activity of the immobilized enzyme preparation was almost invariable after 36 h according to the data from the Fig. 8. What' more, hNFs has the 87.8% relative catalytic activity after 6 times cycle. In the organic-water medium, the hNFs enzyme preparation presents super stability in the synthetic reaction.

### 3.6 Substrate scope of hNFs-catalyzed promiscuous Knoevenagel condensation

The generality and scope of hNFs as catalyst for promiscuous catalysis has been tested on a few aldehydes and methylene compounds (Table 2). Catalytic synthetic activities were also compared between hNFs and free papain under the same reaction conditions and substrates. As shown in Table 1, the yield of product 3 was 46% when catalyzed by hNFs, while nearly no product can be detected when free papain as catalyst. The result of product 4 was similar to product 3, which can be explained by important roles of Cu^2+^ in promiscuous catalysis.^11^ The electronic properties of substituents had some impacts on reaction yields. α-β-Unsaturated aromatic aldehydes with strong electron-withdrawing substituents demonstrated higher yields than those containing strong electron-donating substituents, contradicting to a reported papain-catalyzed Knoevenagel reactions.^21^ This inconsistence is possibly caused by special catalytic effect of hNFs. The yield of product used papain as catalyst was better than hNFs when the substituent group of unsaturated aromatic aldehydes was electrophilic and acetylacetone was substrate. The yield of hNFs was better when the substrate was 3-phenylazoacetylacetone. Control experiments have also been performed with Cu_3_(PO_4_)_2_ without enzyme as catalyst. No product can be detected, so we speculated that Cu^2+^ may concert catalysis with papain.^6,34^

**Table 2:** The hNFs-catalyzed promiscuous reaction of other aldehydes and methylene^a^

Number	R_1_	R_2_	Products	Yield^b^ (%)-papain	Yield^b^ (%)-hNFs
1	C_6_H_4_	Me	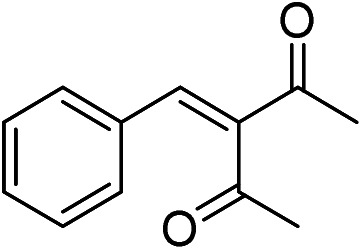	26	33
2	2-NO_2_C_6_H_4_	Me	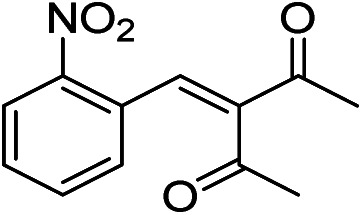	55	53
3	4-ClC_6_H_4_	Me	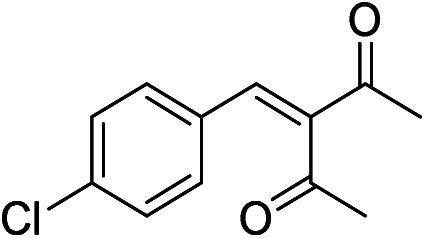	8	9
4	CH_3_–C_6_H_4_	Me	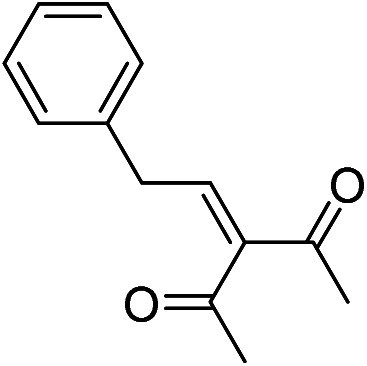	Trace	46
5	–C_6_H_4_	–C_6_H_4_	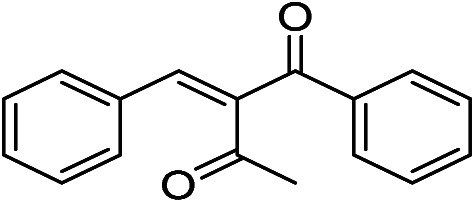	3	21
6	2-NO_2_C_6_H_4_	–C_6_H_4_	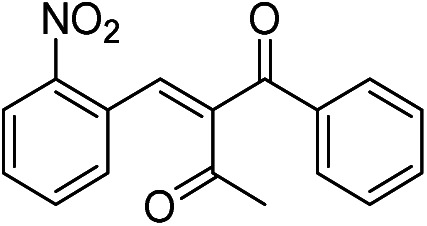	44	50
7	4-BrC_6_H_4_	–C_6_H_4_	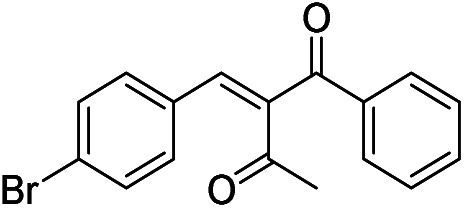	16	9
8	CH_3_–C_6_H_4_	–C_6_H_4_	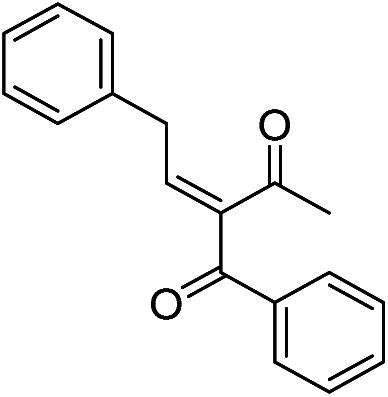	39	47
9	Me	Me	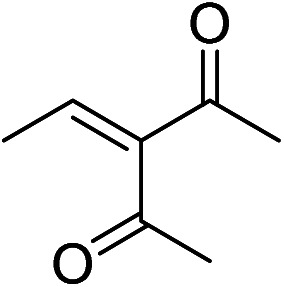	9	15
10	Et	Me	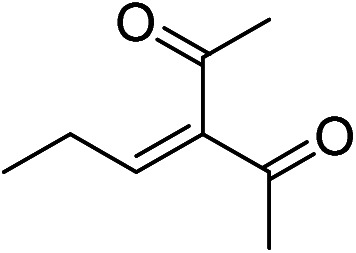	39	47

a Conditions: aldehyde (2 mmol), methylene (2.4 mmol), hNFs/papain (150 mg), DI water (1.25 mL), DMSO (3.75 mL) at 60 °C for 24 h.

b Yield of the isolated product after purification.

### 3.7 Proposed catalytic mechanism for the hNFs-catalyzed Knoevenagel reaction

**Scheme 1:**
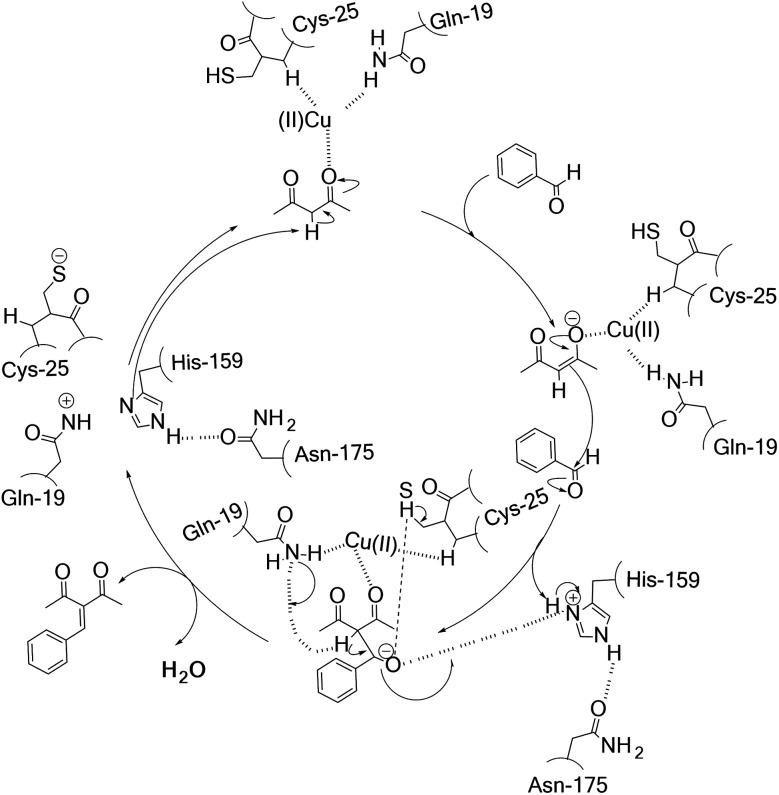
Proposed mechanism for the hNFs-catalyzed Knoevenagel condensation.

His-159, Gln-19, Asn-175 and Cys-25 all belong to single polypeptide chain of papain that involved 212 residues. The shape of papain molecule was seem consist of two hemispheres. There was a pretty deep groove between two hemispheres. The active site was located at bottom of the groove, in which Cys-25 and His-159 were on both side. Based on the predictions by Wen and his coworkers regarding papain^21^ and the role of Cu(ii) site played in the enzyme biocatalysis proposed by Solomon,^35^ we proposed a potential mechanism of hNFs-catalyzed Knoevenagel reaction in the presence of Cu^2+^ (Scheme 1). In the catalysis, substrate molecule binds to the copper site as a monodentate ligand *via* the carbonyl and then formed the Cu(ii)–substrate complex. At the beginning, the proton on the substrate acetylacetone is attacked by His-159 and the carbonyl oxygen on acetylacetone attacked by carbon–oxygen double bond forming an enolate anion, stabilized by the oxyanion hole formed by Gln-19 and Cys-25 (ref. 16) and the His-159 is stabilized by Asn-175. Subsequently, another substrate accepts the proton from carbon–carbon double bond and simultaneously connected the enolate anion with the formation of a carbon–carbon bond. Lastly, the dehydration of the resulted compound took place under the catalysis of Gln-19, Cys-25, and His-159, leading to Knoevenagel product. Meanwhile, the Cys–His ion pair formed which was stabilized by Cu^2+^*via* keeping the imidazole ring of His-159 in a favorable orientation.

When solely used in the chemical catalysis reaction, Cu^2+^ ion often plays an important role due to the coordination with N atom. Jiang and his coworker^36^ have introduced Cu^2+^ to the *p*-zazcrown[*N*,*S*,*O*]-styryl-modified boron-phenylpyrrin compound synchronously possessing a diethylamino receptor (C-BPP-A). Cu^2+^ could coordinated with the *p*-zazcrown[*N*,*S*,*O*] moiety of C-BPP-A, forming the Cu^2+^-BPP-A complex and leading to the significant enhancement in fluorescence emission at 621 nm. Furthermore, if magnetic performance as report^37^ will be attached to hNFs, this enzyme preparation would be applied even more widely in the promiscuous catalysis.

### 3.8 MS and NMR characterization

**Fig. 9:**
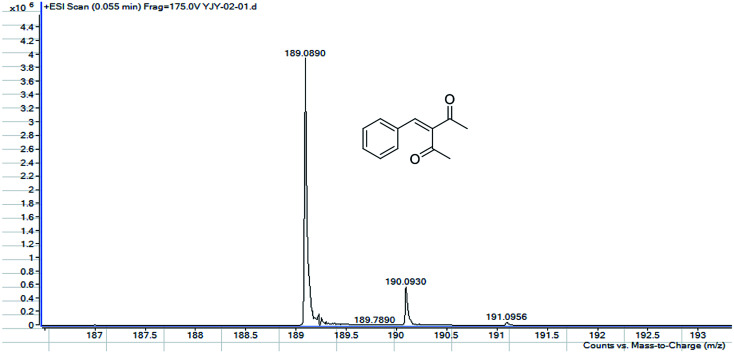
MS of 3-benzylidenepentane-2,4-dione catalyzed by hNFs.

MS, ^1^H NMR, and ^13^C NMR have been used to characterize the obtained products, respectively. Fig. 9 shows the MS of 3-benzylidenepentane-2,4-dione catalyzed by hNFs in DMSO containing 25% water for 24 h. The molecular weight of the product was 188.23 and the ion peak was 189.07 were indicated in this figure. The ion peak is considered to be the M + H peak, consistent with molecular weight of the product.

**Fig. 10:**
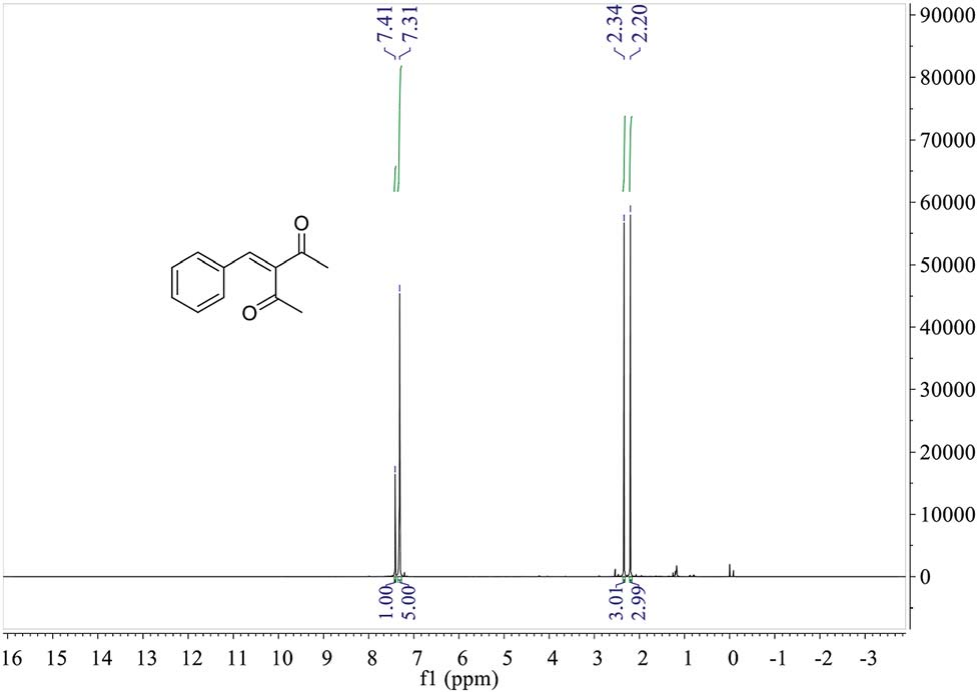
^1^H NMR of 3-benzylidenepentane-2,4-dione catalyzed by hNFs.

The ^1^H NMR spectrum of 3-benzylidenepentane-2,4-dione catalyzed by hNFs (DMSO containing 25% water for 24 h), along with peak positions and assignments, was displayed in Fig. 10. The data is: *δ* 7.41 (s, 1H), 7.31 (m, 5H), 2.34 (s, 3H), 2.20 (s, 3H).

**Fig. 11:**
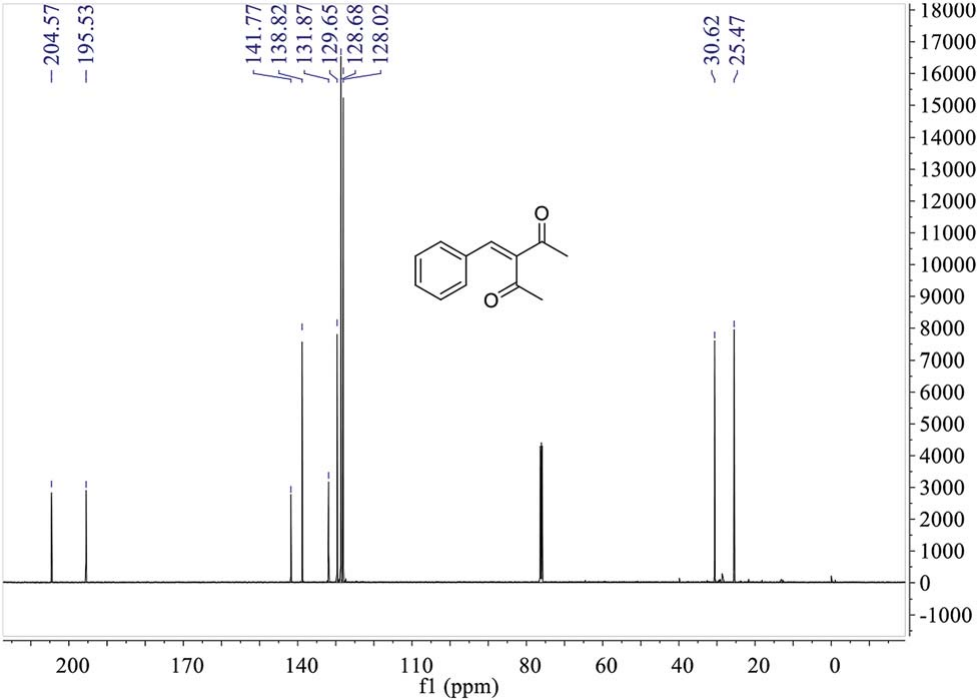
^13^C NMR of 3-benzylidenepentane-2,4-dione catalyzed by hNFs.

The ^13^C NMR spectrum of 3-benzylidenepentane-2,4-dione catalyzed by hNFs in DMSO containing 25% water for 24 h, along with peak positions and assignments, was displayed in Fig. 11. The data is as following: *δ* 204.57 (s), 195.53 (s), 141.77 (s), 138.82 (s), 131.87 (s), 129.65 (s), 128.68 (s), 128.02 (s), 30.62 (s), 25.47 (s).

## 4. Conclusion

In summary, we developed a simple method with mild condition to prepare a concerted papain@Cu_3_(PO_4_)_2_ catalyst for Knoevenagel condensation. In this hybrid nanoflowers catalyst, Cu^2+^ from Cu_3_(PO_4_)_2_ crystals stabilize the structure of papain protein and accelerates papain-catalyzed Knoevenagel condensation. Moreover, the large size of hybrid nanoflowers could be easily recycled, avoiding tedious separation procedures and remained metal Cu^2+^ in liquid mixture. Further, metal Cu^2+^ in hNFs can also be recycled using NH_3_·H_2_O and phosphoric acid if hNFs lose its activity reaction, avoiding potential environment pollution. This facile preparation of hNFs green biocatalyst also implies simple applicability in organic synthesis by promiscuous catalysis.

## Conflicts of interest

There are no conflicts to declare.

## Supplementary Material

RA-008-C7RA12940H-s001
