# Supplementary material for: Efficient promiscuous Knoevenagel condensation catalyzed by papain confined in Cu3(PO4)2 nanoflowers
Source: RSC Adv. 2018 Jan 9;8(5):2357–64. doi: 10.1039/c7ra12940h (PMC9077389; doi:10.1039/c7ra12940h)

Electronic Supplementary Material (ESI) for RSC Advances.
This journal is © The Royal Society of Chemistry 2017

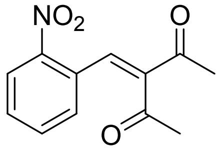

^{1}H NMR (500 MHz, $CDCl_3$) δ 8.16 (dd, J = 8.2, 1.2 Hz, 1H), 7.85 (s, 1H), 7.59 (m, J = 7.5, 1.1 Hz, 1H), 7.55 – 7.49 (m, 1H), 7.32 (d, J = 7.6 Hz, 1H), 2.41 (s, 3H), 2.05 (s, 3H).

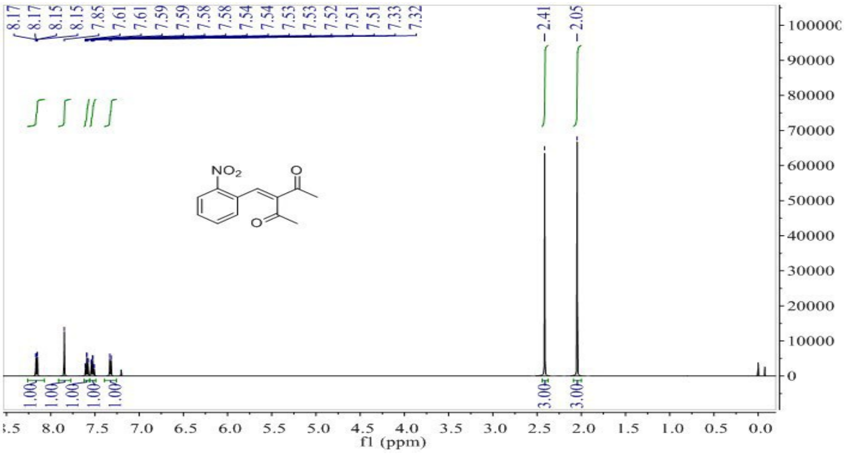

^{13}C NMR (126 MHz, $CDCl_3$) δ 201.78 (s), 195.30 (s), 146.01 (s), 143.19 (s), 136.76 (s), 133.10 (s), 130.17 (s), 129.52 (s), 128.92 (s), 124.24 (s), 30.70 (s), 25.75 (s).

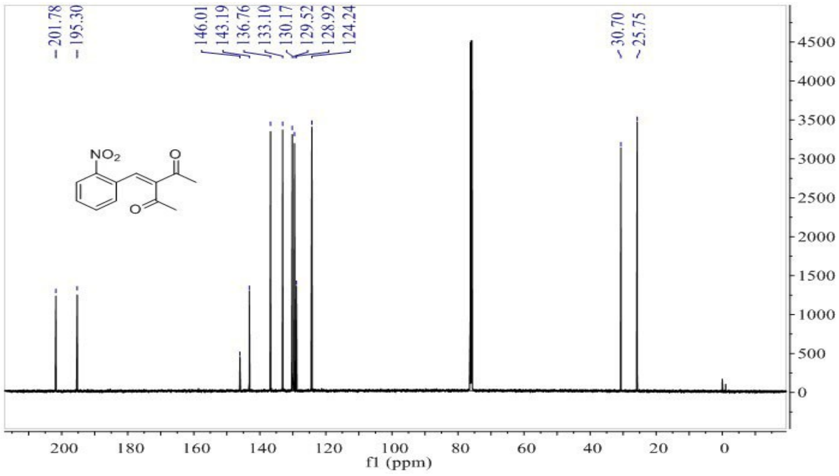

LC-MS

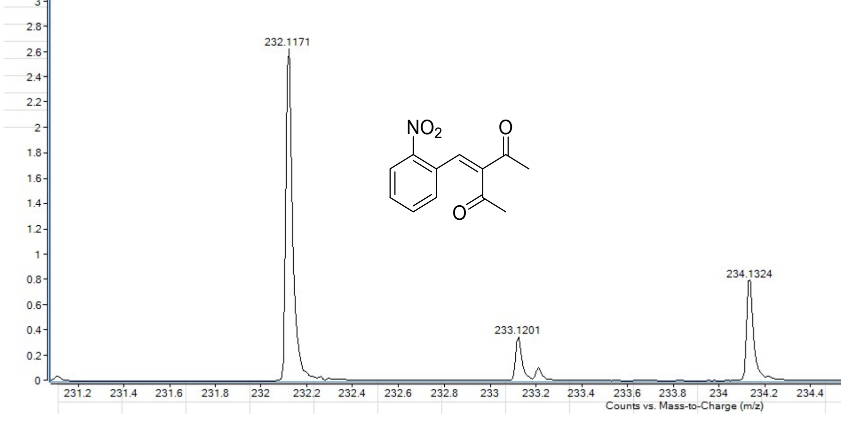

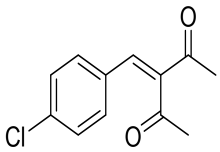

^{1}H NMR (500 MHz, $CDCl_3$) δ 7.35 (s, 1H), 7.31 (s, 1H), 7.28 (d, J = 4.7 Hz, 1H), 7.27 (s, 1H), 7.25 (s, 1H), 2.35 (s, 3H), 2.21 (s, 3H).

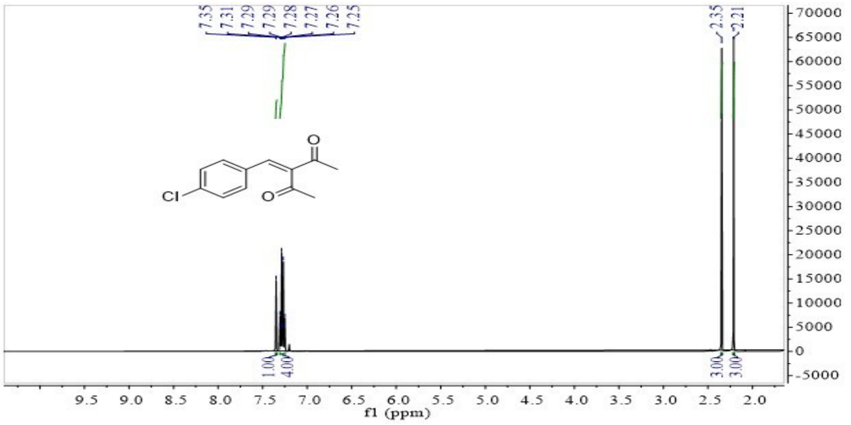

^{13}C NMR (126 MHz, $CDCl_3$) δ 204.29 (s), 195.27 (s), 137.22 (s), 130.35 (s), 129.88 (s,3), 128.34 (s,3), 30.64 (s), 25.49 (s).

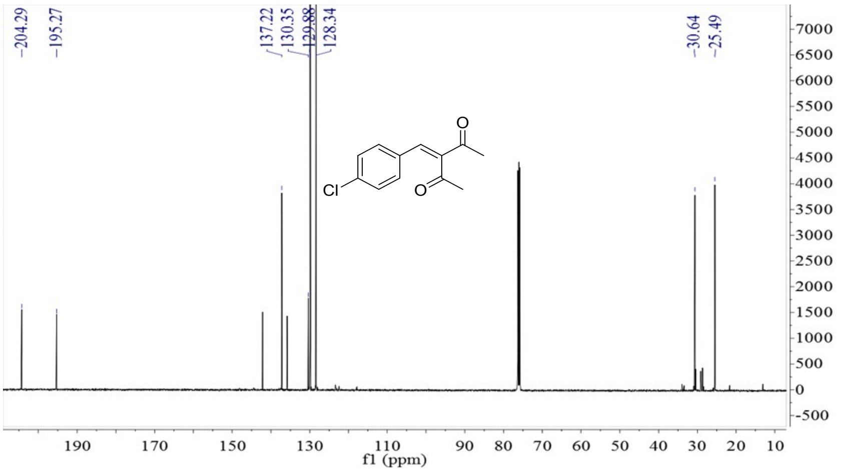

LC-MS

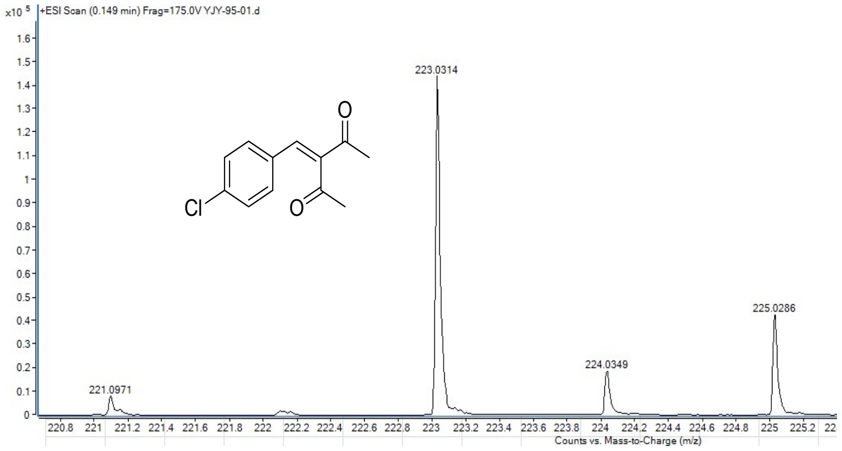

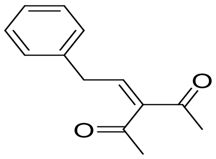

^{1}H NMR (500 MHz, $CDCl_3$) δ 8.18 (s, 2H), 7.49 (s, 2H), 7.43 (s, 2H), 7.21 (s, 1H), 2.39 (s, 3H), 2.22 (s, 3H), 1.18 (s, 2H).

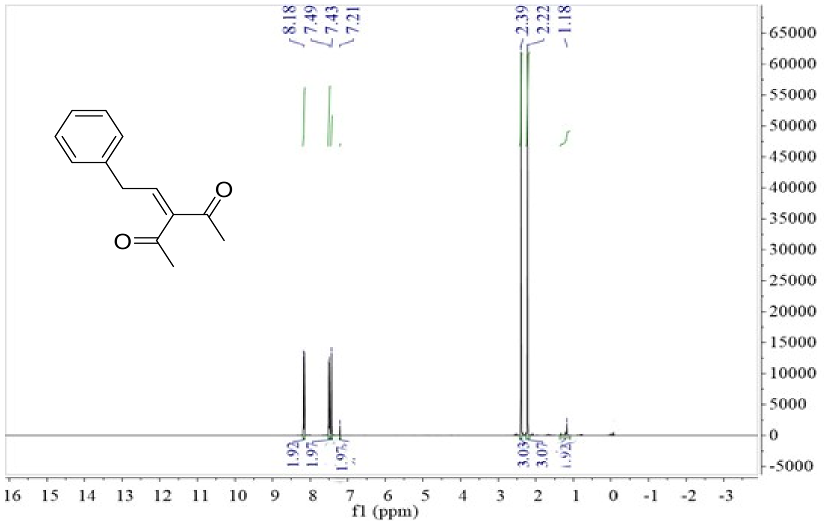

^{13}C NMR (126 MHz, CDCl3) δ 201.23 (d, J = 26.3 Hz), 141.97 (s), 139.29 (s), 129.54 (dd, J = 18.2, 13.3 Hz), 31.76 (s), 29.75 (d, J = 5.3 Hz).

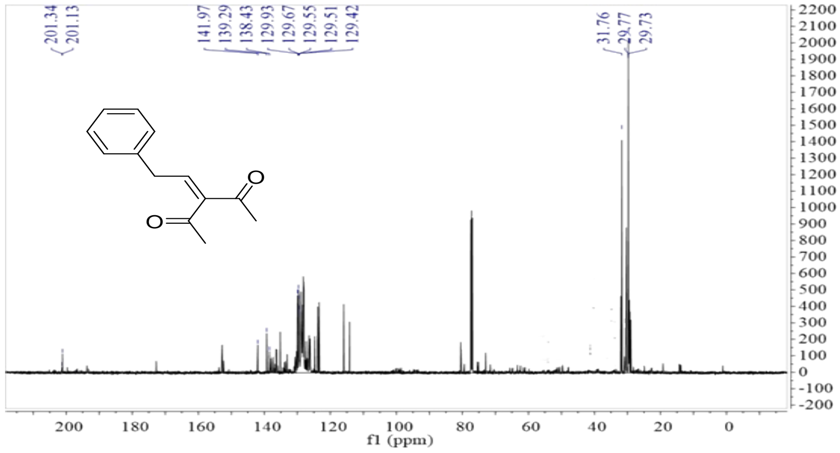

LC-MS

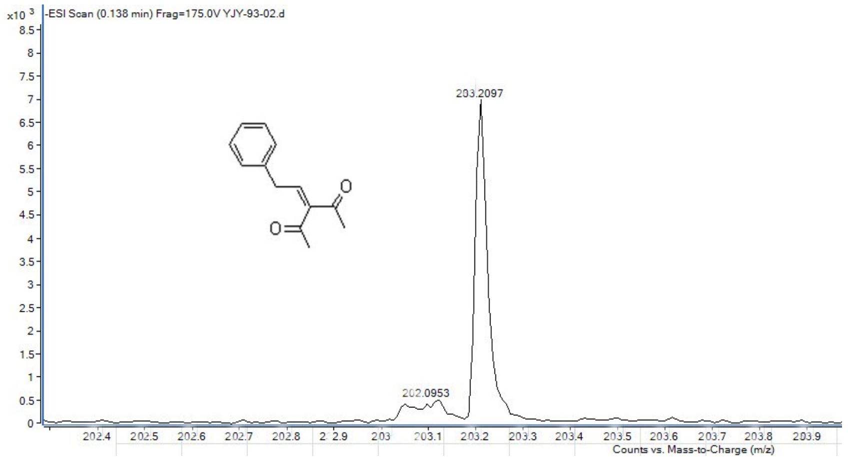

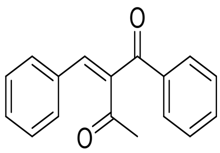

^{1}H NMR (500 MHz, $CDCl_3$) δ 7.82 (dd, J = 8.3, 1.1 Hz, 2H), 7.69 (s, 1H), 7.33 – 7.20 (m, 1H), 7.12 (ddd, J = 14.4, 7.7, 6.2 Hz, 4H), 5.14 (s, 1H), 2.29 (s, 3H).

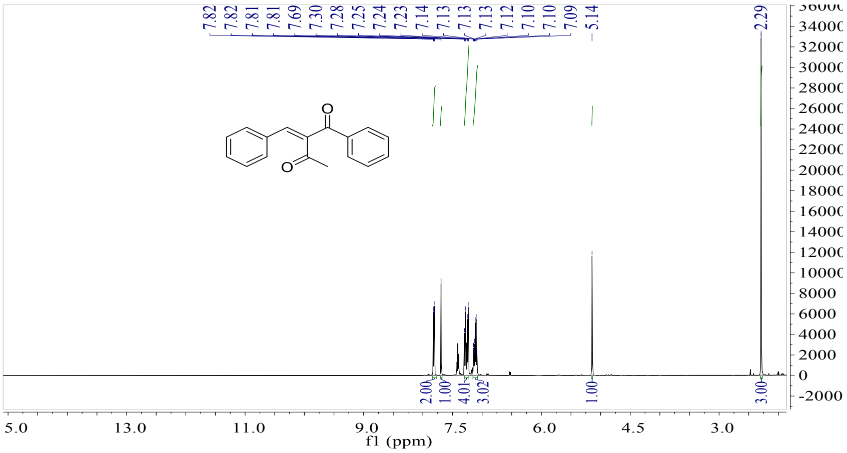

^{13}C NMR (126 MHz, $CDCl_3$) δ 201.13(s), 199.66(s), 152.79(s), 135.18(s), 134.00(s), 133.60(s), 133.08(s), 128.99(s), 128.91(s), 128.87(s), 128.83(s), 128.48(s), 128.38(s), 128.18(s), 128.15(s), 128.12(s), 127.99(s).

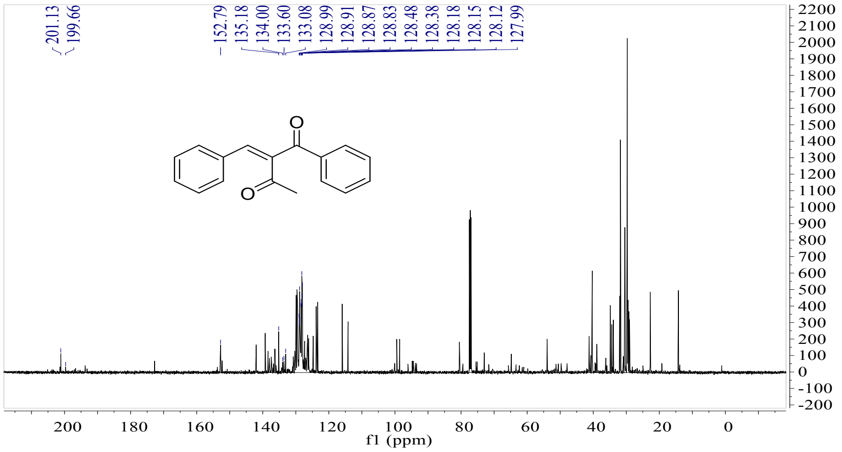

LC-MS

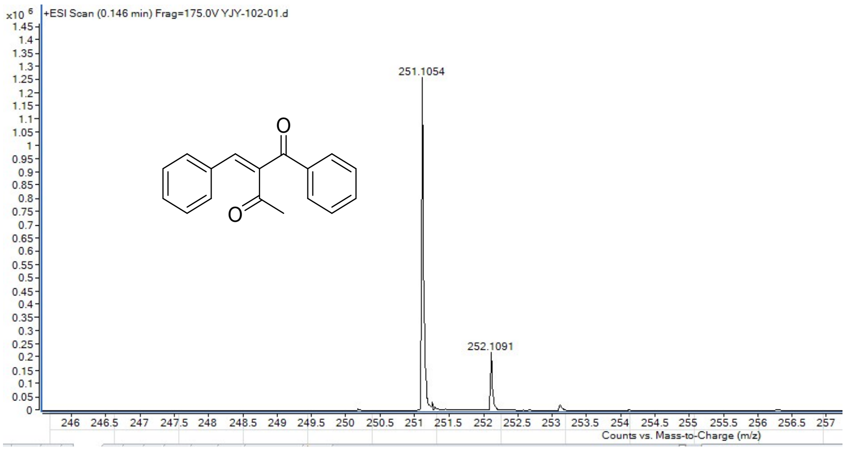

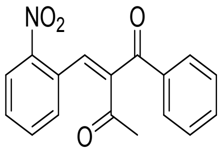

^{1}H NMR (500 MHz, $CDCl_3$) δ 8.19 (s, 1H), 8.05 (d, J = 8.2 Hz, 1H), 7.83 – 7.75 (m, 2H), 7.49 – 7.36 (m, 4H), 7.31 (t, J = 7.7 Hz, 2H), 2.49 (s, 3H).

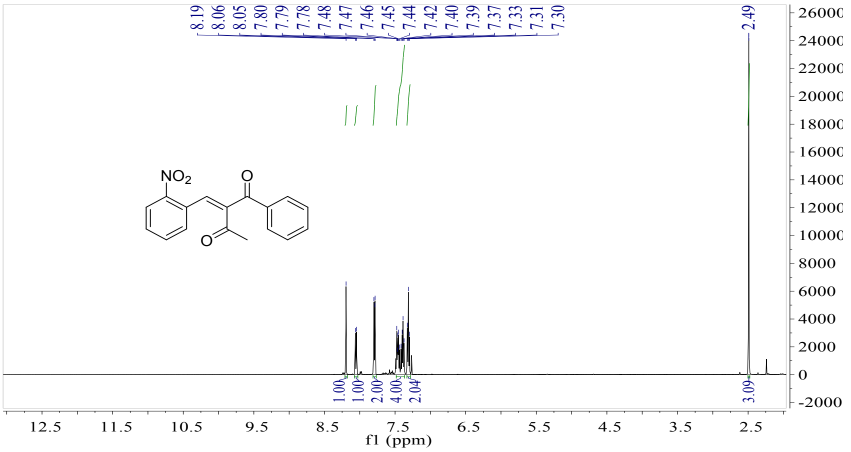

^{13}C NMR (126 MHz, $CDCl_3$) δ 195.97 (s), 195.52 (s), 147.02 (s), 141.49 (s), 138.53 (s), 136.19 (s), 134.09 (s), 133.93 (s), 131.41 (s), 130.30 (s), 130.09 (s), 129.78 (s), 128.95 (s), 128.79 (s), 125.01 (s), 27.37 (s).

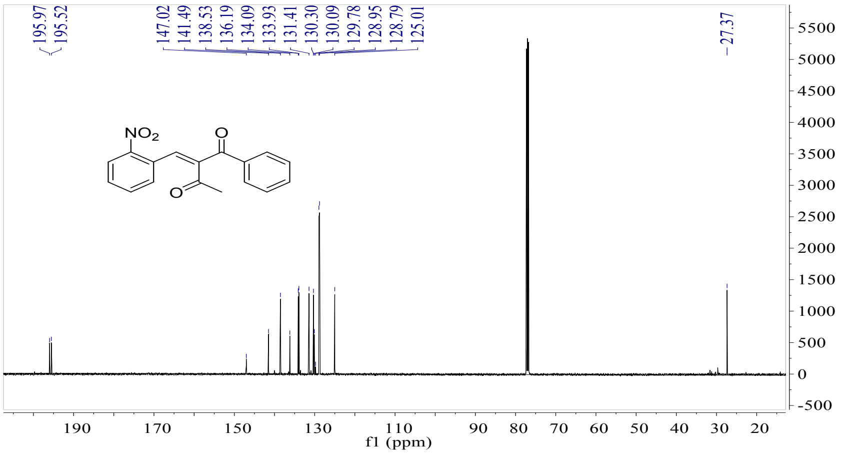

LC-MS

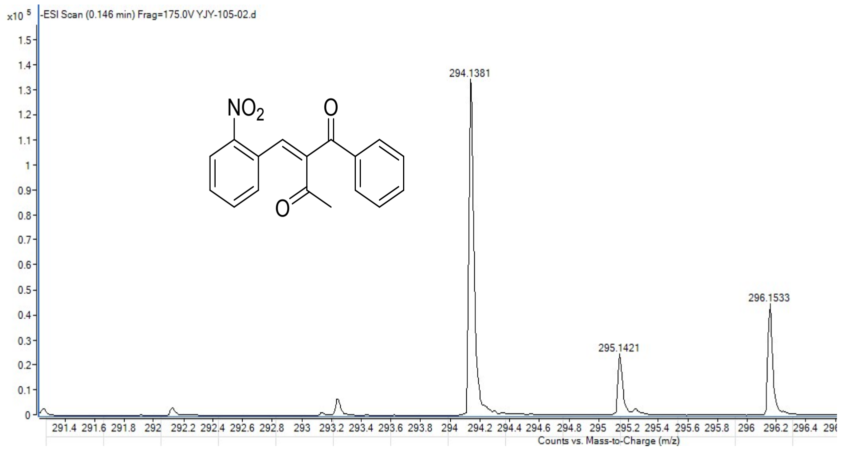

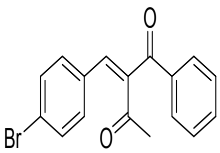

^{1}H NMR (500 MHz, $CDCl_3$) δ 7.81 (dd, *J* = 8.3, 1.1 Hz, 2H), 7.62 (s, 1H), 7.44 (d, *J* = 7.4 Hz, 1H), 7.31 (t, *J* = 7.8 Hz, 2H), 7.24 (d, *J* = 8.6 Hz, 2H), 7.10 (d, *J* = 8.5 Hz, 2H), 2.29 (s, 3H).

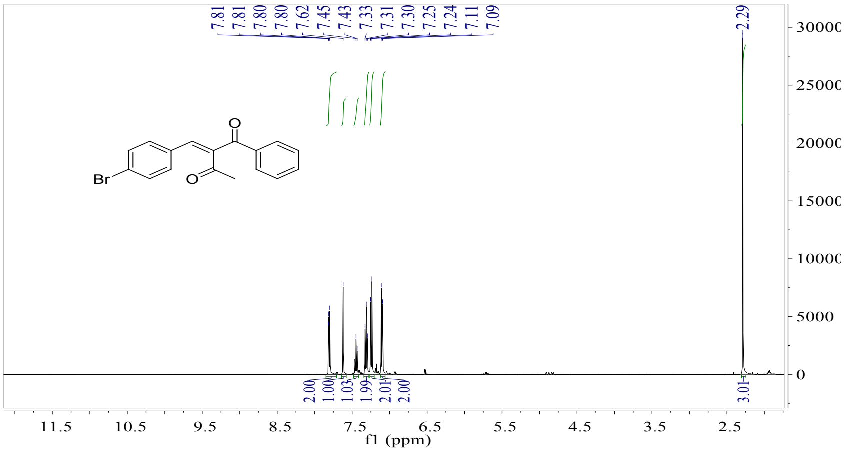

^{13}C NMR (126 MHz, $CDCl_3$) δ 197.79 (s), 195.73 (s), 140.13 (s), 139.74 (s), 135.77 (s), 134.39 (s), 132.12 (s), 131.78 (s), 131.62 (s), 129.15 (s), 129.09 (s), 125.11 (s), 31.68 (s), 29.71 (s), 29.67 (s), 29.66 (s), 27.27 (s).

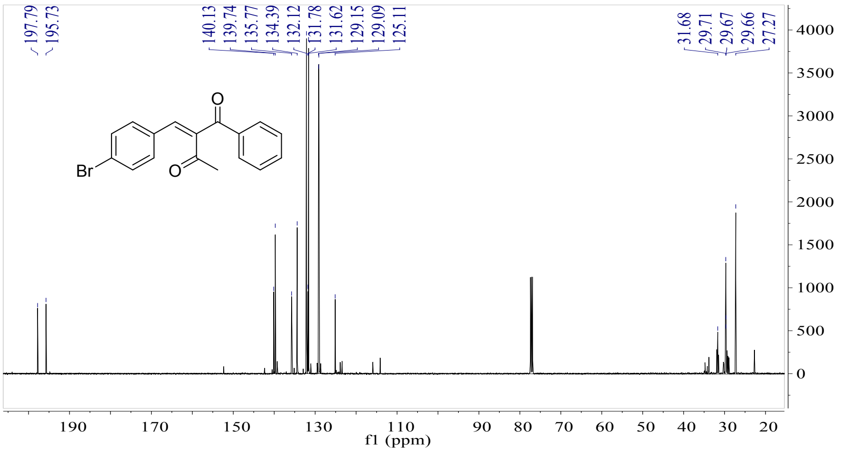

LC-MS

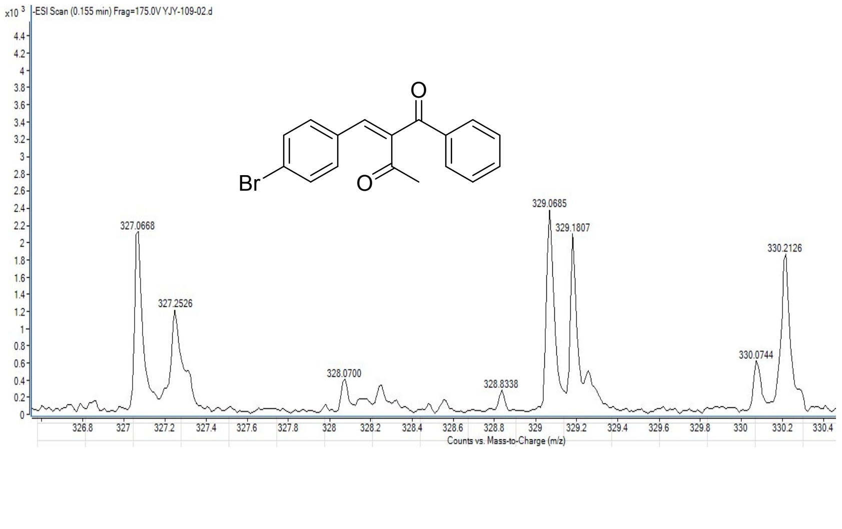

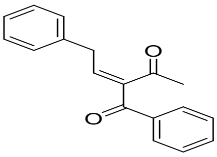

^{1}H NMR (500 MHz, $CDCl_3$) δ 8.19 (s, 1H), 8.05 (s, 1H), 7.78 (d, J = 1.1 Hz, 2H), 7.56 – 7.15 (m, 6H), 2.83 – 1.93 (m, 3H), 1.42 (d, J = 4.0 Hz, 2H).

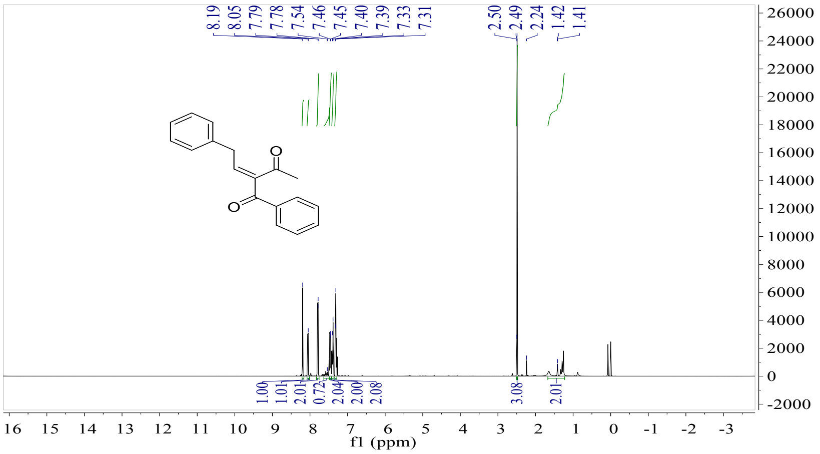

^{13}C NMR (126 MHz, $CDCl_3$) δ 195.97(s), 195.52(s), 147.02(s), 141.49(s), 138.53(s), 136.19(s), 134.09(s), 133.93(s), 133.64(s), 131.41(s), 130.30(s), 130.09(s), 129.78(s), 128.95(s), 128.79(s), 125.01(s), 29.70(s), 27.37(s).

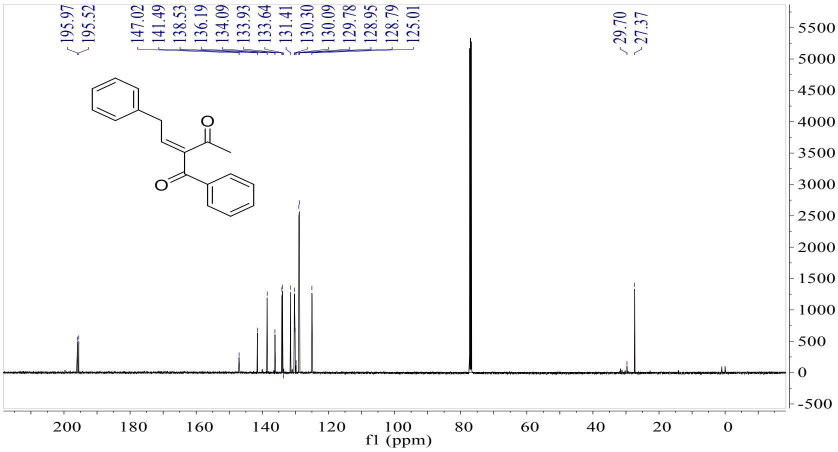

LC-MS

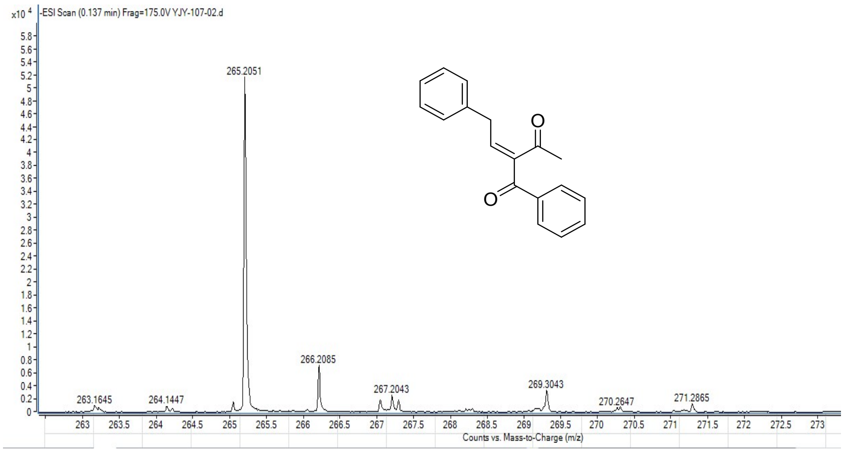

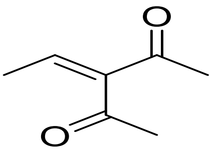

^{1}H NMR (500 MHz, $CDCl_3$) δ 3.61 (td, J = 6.5, 2.0 Hz, 1H), 2.32 – 2.00 (m, 9H).

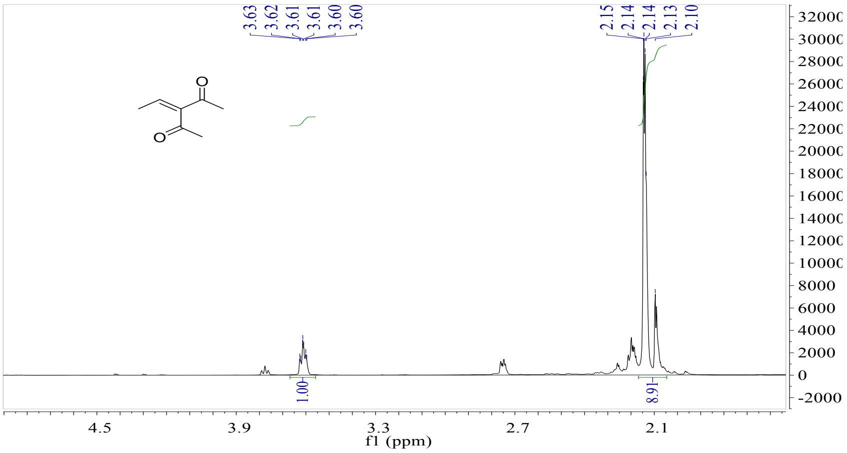

^{13}C NMR (126 MHz, $CDCl_3$) δ 202.71 (s), 190.93 (s), 29.36 (s), 28.61 (s), 25.16 (s), 23.88 (s), 22.24 (s).

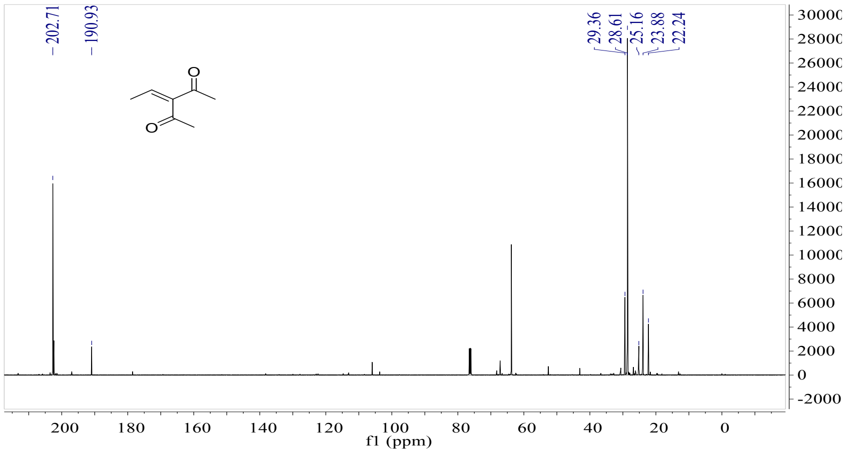

LC-MS

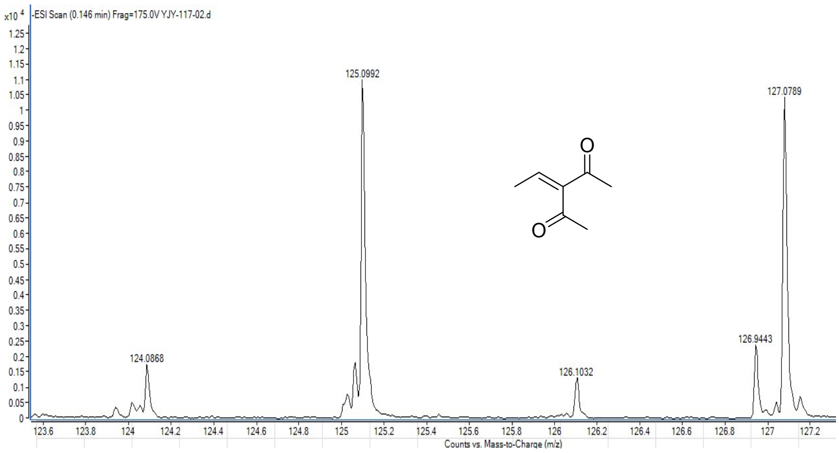

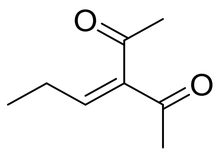

^{1}H NMR (500 MHz, $CDCl_3$) δ 3.60 (s, 1H), 2.36 – 1.92 (m, 10H), 1.23 (s, 1H).

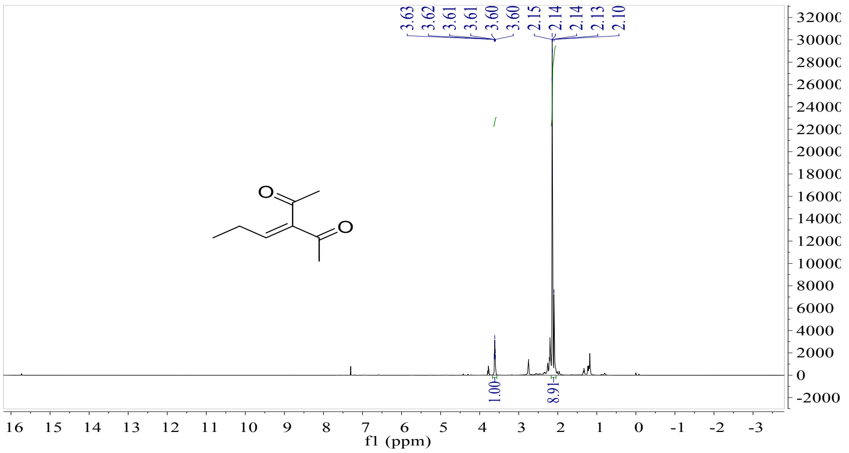

^{13}C NMR (126 MHz, $CDCl_3$) δ 202.71(s), 202.37(s), 190.93(s), 105.96(s), 29.36(s), 28.61(s), 23.88(s), 22.24(s)

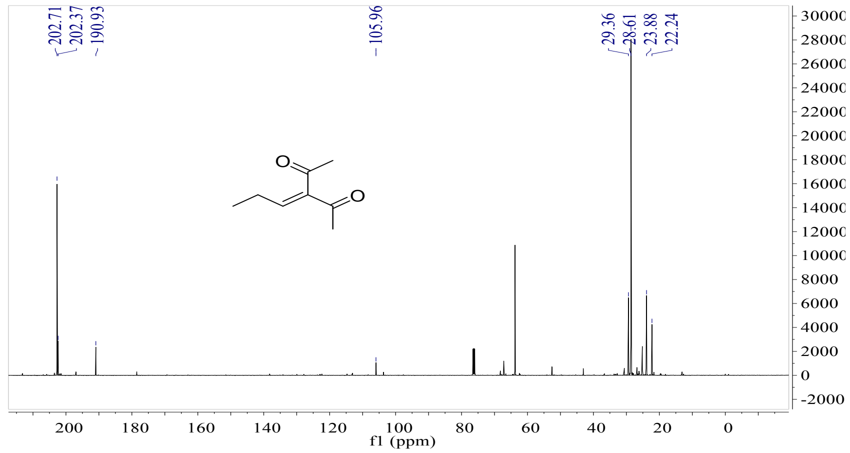

LC-MS

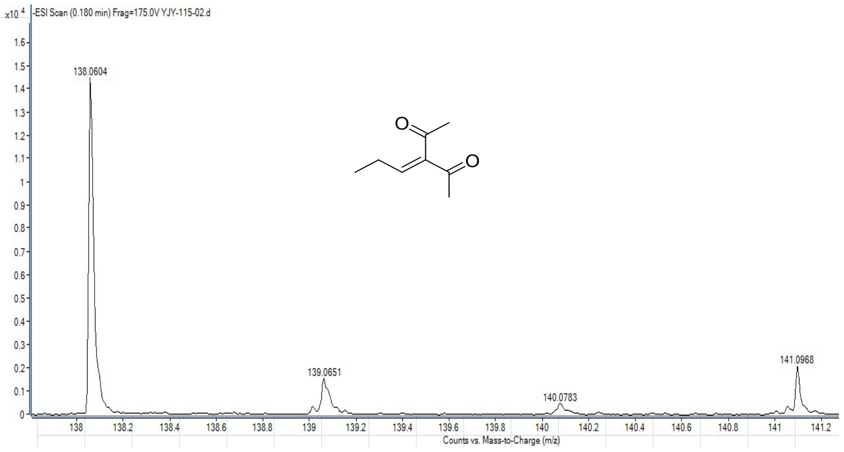

Supplement: RA-008-C7RA12940H-s001 [file RA-008-C7RA12940H-s001.pdf]
